# Misoprostol combined with oxytocin versus oxytocin alone in improving cervical Bishop score during term induction of labor: A retrospective analysis

**DOI:** 10.1097/MD.0000000000047349

**Published:** 2026-02-13

**Authors:** Zehua Zheng, Yang Feng, Wei Yin, Dongfei Guo, Yanli Pei, Qingran Kong, Xuefei Lu

**Affiliations:** a Department of Obstetrics, Central Hospital of Ningcheng County, Chifeng, Inner Mongolia, China; b Perinatal Health Care Center, Central Hospital of Ningcheng County, Chifeng, Inner Mongolia, China.

**Keywords:** Bishop score, induction of labor, misoprostol, oxytocin, term pregnancy

## Abstract

This study aims to investigate the effect of misoprostol combined with oxytocin versus oxytocin alone on improving the cervical Bishop score during term labor induction and its impact on delivery outcomes. A retrospective analysis was conducted on 200 pregnant women who underwent term labor induction and met the inclusion criteria at Ningcheng Central Hospital, Chifeng, between Decemeber 2024 and May 2025. According to different clinical medication regimens, they were divided into a control group (oxytocin alone, n = 100) and a study group (misoprostol combined with oxytocin, n = 100). Baseline data, improvements in cervical Bishop score, labor-related indicators, and delivery outcomes were collected for both groups, and subgroup analyses were performed. There were no statistically significant differences in baseline data between the 2 groups (*P* > .05). After 12 hours of intervention, the improvement in Bishop score was significantly greater in the study group compared with the control group (Δ Bishop score 3.5 ± 1.2 vs 2.4 ± 1.0, *P* < .001), with a higher overall effective rate (87.0% vs 71.0%, *P* = .012). The study group also showed significantly shorter times from medication to onset of labor (8.4 ± 2.9 vs 11.8 ± 3.5 hours) and for the first stage of labor (7.2 ± 2.8 vs 9.6 ± 3.2 hours) (both *P* < .001). The vaginal delivery rate in the study group was 86.0%, higher than 72.0% in the control group (*P* = .015). Subgroup analysis indicated that the combined regimen had advantages in both primiparas and multiparas, as well as in women at 37 to 39 weeks or ≥40 weeks of gestation, with more pronounced benefits in primiparas and those at 37 to 39 weeks. Misoprostol combined with oxytocin during term labor induction can significantly improve cervical ripening, shorten the time from medication to labor onset and the first stage of labor, and increase the vaginal delivery rate, showing high clinical application value.

## 1. Introduction

Induction of labor is a common clinical procedure in obstetrics, mainly indicated for pregnancy complications, post-term pregnancy, premature rupture of membranes, or other conditions requiring the timely termination of pregnancy. According to data from the World Health Organization, ~18% to 25% of pregnancies worldwide require induction of labor each year.^[1]^ In developed countries, induction rates can reach 20% to 30%, whereas in developing countries, the rates are relatively lower. However, with changes in maternal age structure and an increasing proportion of high-risk pregnancies, the demand for induction is gradually rising.^[2]^ In China, the clinical demand for induction has also increased in recent years due to factors such as advanced maternal age, gestational diabetes mellitus, and hypertensive disorders of pregnancy.^[3]^ The choice of a safe and effective induction method has thus become a major clinical focus.

One of the key determinants of successful induction is cervical maturity. When the cervical Bishop score is low, induction often has a high failure rate and is associated with an increased risk of cesarean delivery, prolonged labor, uterine inertia, postpartum hemorrhage, and neonatal distress.^[4,5]^ Therefore, improving cervical conditions and enhancing the success rate of induction are crucial for optimizing maternal and neonatal outcomes.

Currently, oxytocin remains the most widely used agent for labor induction. It induces labor by promoting uterine contractions and is particularly effective in women with favorable cervical conditions. However, in women with a low Bishop score, oxytocin alone is often insufficient, leading to weak uterine contractions or induction failure.^[6]^ To compensate for oxytocin’s limitations in cervical ripening, prostaglandin agents have been introduced into clinical practice.

Misoprostol, a prostaglandin E1 analogue, exerts dual effects by softening the cervix and stimulating uterine contractions. It is chemically stable, inexpensive, and easy to administer orally or vaginally. Thus, it has been recommended by both the World Health Organization and the International Federation of Gynecology and Obstetrics as one of the first-line agents for the induction of labor.^[7,8]^ Numerous randomized controlled trials and systematic reviews abroad have shown that misoprostol is effective in improving cervical ripening, shortening labor duration, and increasing vaginal delivery rates.^[9]^ However, due to interindividual variability in drug response, misoprostol may also cause risks such as uterine hyperstimulation and fetal heart rate abnormalities, necessitating careful consideration of dosage and administration in clinical use.

In recent years, a combined strategy of misoprostol and oxytocin has been proposed. The theoretical basis lies in using misoprostol first to improve cervical conditions, followed by oxytocin to induce or maintain effective contractions, thereby achieving a dual optimization of the induction process. Existing studies suggest that this combination regimen is superior to oxytocin alone in improving cervical maturity, shortening the time from medication to labor onset and the first stage of labor, and enhancing induction success rates.^[10,11]^ However, most of these studies have been single-center with small sample sizes, some focusing on post-term or high-risk pregnancies, and there remains a lack of systematic retrospective analysis specifically targeting term pregnancies.

In China, clinical reports on the combined use of misoprostol and oxytocin are still limited, and findings remain inconsistent. Some studies have demonstrated a significant reduction in cesarean section rates with the combined regimen, while others have suggested only limited benefits in improving vaginal delivery rates.^[12]^ Moreover, most studies have not stratified by parity or gestational age, limiting their clinical applicability. Therefore, further research based on real-world clinical data is needed to clarify the target population and clinical value of the combined regimen.

Based on this, the present study employed a retrospective cohort design, collecting cases of term pregnant women who underwent induction of labor at Ningcheng Central Hospital, Chifeng, between September 2022 and May 2025. The study compared misoprostol combined with oxytocin versus oxytocin alone in terms of cervical Bishop score improvement, labor-related indicators, and delivery outcomes, with additional subgroup analyses by parity and gestational age. The findings aim to provide evidence-based support for developing more scientifically sound labor induction protocols in clinical practice.

## 2. Materials and methods

### 2.1. Study design

This study was approved by the Ethics Committee of Central Hospital of Ningcheng County. This study adopted a retrospective cohort design, collecting clinical records of pregnant women who underwent term induction of labor at Ningcheng Central Hospital, Chifeng, between Decemeber 2024 and May 2025. According to predefined inclusion and exclusion criteria, eligible participants were categorized into 2 groups based on their actual clinical medication regimen: an oxytocin-only group and a misoprostol combined with oxytocin group. Baseline characteristics between the 2 groups were matched and balanced. A total of 200 women were ultimately included, with 100 cases in the control group (oxytocin alone) and 100 cases in the study group (misoprostol combined with oxytocin).

#### 2.1.1. Inclusion criteria

Age 23 to 33 years; gestational age between 37 + 3 and 41 + 1 weeks, singleton pregnancy, normal fetal presentation, intact membranes; indications for induction of labor, including oligohydramnios, hypertensive disorders of pregnancy (with stable blood pressure and eligible for vaginal delivery), or gestational diabetes mellitus (with well-controlled blood glucose and able to tolerate vaginal delivery); cervical Bishop score < 6; and complete medical records.

#### 2.1.2. Exclusion criteria

Contraindications to vaginal delivery include cephalopelvic disproportion, abnormal fetal presentation, premature rupture of membranes, placenta previa (complete or partial), placental abruption, or fetal distress; history of allergy to misoprostol or oxytocin; and previous uterine surgery (e.g., cesarean section, cervical surgery).

### 2.2. Intervention protocols

#### 2.2.1. Control group (n = 100)

Labor induction was performed using low-dose oxytocin intravenous infusion. After admission to the labor ward, an oxytocin challenge test was conducted. If the result was negative, oxytocin infusion was initiated. A total of 2.5 U oxytocin was diluted in 500 mL of 0.9% sodium chloride solution and administered via intravenous drip at an initial rate of ~8 drops/min. The infusion rate was adjusted every 15 to 30 minutes according to uterine contractions, with each adjustment increasing by 4 drops/min until regular contractions occurred or the maximum infusion rate of 40 drops/min was reached. Throughout the procedure, continuous monitoring of fetal heart rate and uterine contractions (including frequency, intensity, duration, intervals, and uterine relaxation) was performed. If uterine hyperstimulation or abnormal fetal heart monitoring was observed, the infusion was immediately discontinued.

#### 2.2.2. Study group (n = 100)

Labor induction was performed using misoprostol combined with oxytocin. After bladder emptying, the patient was placed in the supine position, and under aseptic conditions, a 25-μg vaginal tablet of misoprostol was inserted into the posterior fornix. Following administration, the patient was required to remain in bed for 30 minutes with fetal heart monitoring. If no regular uterine contractions occurred after 6 hours, an additional 25-μg dose could be administered (maximum cumulative dose not exceeding 50 μg). If labor had not commenced ≥4 hours after the last administration, low-dose oxytocin intravenous infusion was initiated following the same protocol as in the control group.

During induction, if uterine hyperstimulation or abnormal fetal heart monitoring occurred as a result of misoprostol, a vaginal examination was performed. When necessary, any incompletely absorbed drug was removed, and tocolytics (e.g., magnesium sulfate) were administered for symptomatic management.

### 2.3. Data collection

All case data in this retrospective study were obtained from the hospital’s electronic medical record system and delivery records to ensure the completeness and reliability of the information.

The collected data included the following aspects:

General demographic data: maternal age, gestational age, gravidity, parity, primiparous or multiparous status, and body mass index.

Admission and pregnancy-related data: cervical Bishop score at admission and the proportion with a score <4, pregnancy complications (hypertensive disorders of pregnancy, gestational diabetes mellitus), estimated fetal weight, and amniotic fluid index.

Indicators of induction and labor process: changes in Bishop score before and after intervention, improvement in Bishop score (Δ Bishop score), and effective rate of cervical ripening.

Labor-related indicators: time from medication to onset of labor, and durations of the first, second, and third stages of labor.

Delivery outcomes: induction success rate (vaginal delivery rate) and cesarean section rate.

### 2.4. Statistical analysis

All statistical analyses were performed using SPSS version 20.0 (SPSS Inc., Chicago) software. Measurement data were expressed as mean ± standard deviation (x̄ ± s). For normally distributed variables, comparisons between groups were performed using the independent samples *t* test, and within-group pre- and post-intervention comparisons were performed using the paired *t* test. For data not conforming to a normal distribution, nonparametric tests were applied. Categorical data were expressed as frequencies and percentages, and intergroup comparisons were performed using the χ² test or Fisher exact test.

Subgroup analysis was conducted using stratification according to parity (primiparous/multiparous) and gestational age (37–39 weeks/≥40 weeks). Interaction tests were further performed to evaluate the potential interaction between intervention methods and stratification factors. All statistical tests were 2-sided, and *P* < .05 was considered statistically significant.

## 3. Results

### 3.1. Comparison of general baseline characteristics between the 2 groups

Baseline characteristics of the eligible pregnant women included in the final analysis were compared between the 2 groups. No statistically significant differences were observed between the control group and the study group with respect to maternal age, gestational age, gravidity, parity, proportion of primiparas, body mass index, Bishop score at admission, or the proportion of cases with Bishop score <4 (all *P* > .05). The mean maternal age was 29.1 ± 2.7 years in the control group and 28.4 ± 2.3 years in the study group. The mean gestational age was 39.1 ± 1.3 weeks in the control group and 38.9 ± 1.1 weeks in the study group. The Bishop scores at admission were 4.0 ± 1.1 and 4.3 ± 1.2, respectively, with no significant difference between groups.

In addition, no significant differences were found between the 2 groups in the incidence of hypertensive disorders of pregnancy (7.0% vs 9.0%), gestational diabetes mellitus (13.0% vs 10.0%), estimated fetal weight, or amniotic fluid index (all *P* > .05; Table 1).

**Table 1:** Baseline characteristics of the 2 groups (x̄ ± s or n/%).

Characteristics	Control group (oxytocin only, n = 100)	Study group (misoprostol + oxytocin, n = 100)	*P*-value
Age (yr)	29.1 ± 2.7	28.4 ± 2.3	.08
Gestational age (wk)	39.1 ± 1.3	38.9 ± 1.1	.17
Gravidity (times)	2.3 ± 1.1	2.0 ± 0.9	.09
Parity (times)	0.8 ± 0.6	0.9 ± 0.5	.42
Primipara, n (%)	57 (57.0)	63 (63.0)	.34
BMI (kg/m²)	24.6 ± 3.1	23.9 ± 2.8	.15
Bishop score at admission	4.0 ± 1.1	4.3 ± 1.2	.07
Bishop score < 4, n (%)	41 (41.0)	37 (37.0)	.56
Gestational hypertension, n (%)	7 (7.0)	9 (9.0)	.61
Gestational diabetes, n (%)	13 (13.0)	10 (10.0)	.51
Estimated fetal weight (g)	3267 ± 455	3198 ± 438	.22
Amniotic fluid index (cm)	12.4 ± 3.6	11.9 ± 3.2	.29

BMI = body mass index.

### 3.2. Improvement in cervical Bishop score

Before intervention, there was no statistically significant difference in Bishop score between the 2 groups (control group: 4.0 ± 1.1 vs study group: 4.3 ± 1.2, *P* = .07). After 12 hours of intervention, Bishop scores in both groups increased significantly compared with baseline, with a greater improvement observed in the study group (control group: 6.6 ± 1.3 vs study group: 7.8 ± 1.4, *P* < .001). The Δ Bishop score indicated that the magnitude of improvement was significantly higher in the study group than in the control group (3.5 ± 1.2 vs 2.4 ± 1.0, *P* < .001; Table 2).

**Table 2:** Comparison of cervical Bishop score improvement between the 2 groups.

Indicators	Control group (oxytocin only, n = 100)	Study group (misoprostol + oxytocin, n = 100)	*P*-value
Bishop score before intervention	4.0 ± 1.1	4.3 ± 1.2	.07
Bishop score after 12 h	6.6 ± 1.3	7.8 ± 1.4	<.001
Δ Bishop score	2.4 ± 1.0	3.5 ± 1.2	<.001
Significant improvement (n, %)	40 (40.0)	58 (58.0)	
Effective (n, %)	31 (31.0)	29 (29.0)	
Ineffective (n, %)	28 (28.0)	13 (13.0)	
Overall effective rate (%)	71.0	87.0	.012

Regarding therapeutic efficacy, the number of markedly effective cases in the study group was 58 (58.0%), higher than 40 (40.0%) in the control group. The number of ineffective cases in the study group was only 13 (13.0%), lower than 28 (28.0%) in the control group. In terms of overall effective rate, the study group was significantly superior to the control group (87.0% vs 71.0%, *P* = .012).

### 3.3. Comparison of labor-related indicators

The time from medication to onset of labor and the duration of the first stage of labor were both significantly shorter in the study group compared with the control group. Specifically, the time from medication to onset of labor was 8.4 ± 2.9 hours in the study group versus 11.8 ± 3.5 hours in the control group (*P* < .001). The duration of the first stage of labor was 7.2 ± 2.8 hours in the study group versus 9.6 ± 3.2 hours in the control group (*P* < .001).

However, no statistically significant differences were observed between the 2 groups in the durations of the second and third stages of labor (second stage: 1.1 ± 0.5 vs 1.2 ± 0.6 hours, *P* = .18; third stage: 7.2 ± 2.0 vs 7.5 ± 2.1 minutes, *P* = .27; Table 3).

**Table 3:** Comparison of labor duration between the 2 groups.

Indicators	Control group (oxytocin only, n = 100)	Study group (misoprostol + oxytocin, n = 100)	*P*-value
Induction-to-labor time (h)	11.8 ± 3.5	8.4 ± 2.9	<.001
First stage of labor (h)	9.6 ± 3.2	7.2 ± 2.8	<.001
Second stage of labor (h)	1.2 ± 0.6	1.1 ± 0.5	.18
Third stage of labor (min)	7.5 ± 2.1	7.2 ± 2.0	.27

### 3.4. Comparison of induction success rate

There was a significant difference in the induction success rate between the 2 groups. The vaginal delivery rate in the study group was 86.0% (86/100), which was significantly higher than 72.0% (72/100) in the control group (χ² = 5.89, *P* = .015; Table 4). Correspondingly, the cesarean section rate in the study group was 14.0%, lower than 28.0% in the control group. These results suggest that misoprostol combined with oxytocin can significantly improve the success rate of induction of labor in term pregnancies.

**Table 4:** Comparison of induction success rate between the 2 groups.

Indicators	Control group (oxytocin only, n = 100)	Study group (misoprostol + oxytocin, n = 100)	χ² value	*P*-value
Vaginal delivery, n (%)	72 (72.0)	86 (86.0)	5.89	.015
Cesarean section, n (%)	28 (28.0)	14 (14.0)		
Induction success rate (%)	72	86		

### 3.5. Subgroup analysis by parity

In the primiparous subgroup, the study group demonstrated superior outcomes compared with the control group in terms of Bishop score improvement, time from medication to onset of labor, and duration of the first stage of labor (all *P* < .05). For example, the mean Δ Bishop score in the study group was 3.7, significantly higher than 2.5 in the control group; the time from medication to onset of labor was ~8.6 hours, shorter than 12.4 hours in the control group. In addition, the vaginal delivery rate was also higher in the study group (87.3% vs 68.4%, *P* = .018).

In the multiparous subgroup, the study group also showed certain advantages. Significant differences were observed in Bishop score improvement and shortened labor duration, although the difference in vaginal delivery rate was not statistically significant (83.8% vs 76.7%, *P* = .39).

Interaction analysis revealed no significant interaction between parity and intervention method (*P* > .05), suggesting that misoprostol combined with oxytocin provided benefits across both primiparous and multiparous women, with more pronounced effects observed in primiparas (Table 5).

**Table 5:** Subgroup analysis by parity.

Indicators	Primipara control (n = 57)	Primipara study (n = 63)	*P*-value	Multipara control (n = 43)	Multipara study (n = 37)	*P*-value	Interaction *P*-value
Δ Bishop score	2.5 ± 1.0	3.7 ± 1.2	<.001	2.6 ± 1.0	3.2 ± 1.1	.02	.12
Induction-to-labor time (h)	12.4 ± 3.4	8.6 ± 2.8	<.001	10.9 ± 3.2	8.2 ± 2.9	.004	.18
First stage of labor (h)	10.1 ± 3.1	7.4 ± 2.7	<.001	8.8 ± 3.0	7.0 ± 2.6	.01	.14
Vaginal delivery, n (%)	39 (68.4)	55 (87.3)	.018	33 (76.7)	31 (83.8)	.39	.09

### 3.6. Subgroup analysis by gestational age

In women with gestational age of 37 to 39 weeks, the study group demonstrated a greater improvement in Bishop score compared with the control group (3.6 vs 2.6, *P* < .001). The time from medication to onset of labor and the duration of the first stage of labor were also significantly shorter in the study group (8.1 vs 11.2 hours; 7.0 vs 9.2 hours, both *P* < .001). The vaginal delivery rate in the study group was 87.7%, higher than 75.8% in the control group (*P* = .045).

In women with gestational age ≥40 weeks, the study group similarly showed advantages in terms of Bishop score improvement and shortened labor duration (both *P* < .01). However, the difference in vaginal delivery rate between the 2 groups did not reach statistical significance (82.9% vs 65.8%, *P* = .12).

Interaction analysis indicated no significant interaction between gestational age and intervention method (*P* > .05), suggesting that the combined regimen had positive effects across different gestational age groups (Table 6).

**Table 6:** Subgroup analysis by gestational age.

Indicators	37–39 weeks control (n = 62)	37–39 weeks study (n = 65)	*P*-value	≥40 weeks control (n = 38)	≥40 weeks study (n = 35)	*P*-value	Interaction *P*-value
Δ Bishop score	2.6 ± 1.0	3.6 ± 1.1	<.001	2.3 ± 1.0	3.4 ± 1.2	.001	.62
Induction-to-labor time (h)	11.2 ± 3.3	8.1 ± 2.7	<.001	12.7 ± 3.6	8.9 ± 3.1	<.001	.21
First stage of labor (h)	9.2 ± 3.0	7.0 ± 2.6	<.001	10.3 ± 3.3	7.6 ± 2.9	<.001	.24
Vaginal delivery, n (%)	47 (75.8)	57 (87.7)	0.045	25 (65.8)	29 (82.9)	0.12	.41

## 4. Discussion

The results of this study indicate that misoprostol combined with oxytocin offers certain advantages over oxytocin alone in promoting cervical ripening, shortening labor duration, and improving the success rate of induction of labor in term pregnancies. As no statistically significant differences were observed in baseline characteristics between the 2 groups, the comparative results may be considered relatively reliable.

In terms of trends, the study group showed more significant improvement in Bishop scores. Although the cervical conditions were comparable between the 2 groups before intervention, after 12 hours, the degree of improvement was greater in the study group, with a relatively higher overall effective rate. This phenomenon may be associated with the pharmacological action of misoprostol, a prostaglandin E1 analogue, which facilitates cervical softening and dilation by promoting collagen degradation and increasing cervical hydration.^[13,14]^ When combined with oxytocin, misoprostol may not only improve cervical conditions but also enhance uterine sensitivity to oxytocin, thereby exerting a synergistic effect.^[15,16]^

Regarding labor-related indicators, this study found that the time from medication to onset of labor and the duration of the first stage of labor were both shortened in the study group, while no significant differences were observed in the durations of the second and third stages. This suggests that the primary effect of misoprostol is concentrated on cervical ripening and the initiation of early labor, with relatively limited influence on the later stages of delivery.^[17]^ From a clinical perspective, such a phased effect may help shorten the latent phase and reduce the risk of dystocia and related complications, particularly in women with unfavorable cervical conditions.

In terms of delivery outcomes, the vaginal delivery rate was higher in the study group, with a corresponding reduction in the cesarean section rate. This trend suggests that the combined regimen may contribute to reducing the need for cesarean intervention. Subgroup analysis further revealed that the advantages of the combined regimen were more pronounced in primiparous women and those at 37 to 39 weeks of gestation. This may be related to poorer cervical conditions in primiparas or greater cervical elasticity at earlier gestational ages, making the cervix more responsive to pharmacological intervention. In contrast, among multiparas or women at ≥40 weeks, although improvements in cervical ripening and shortened labor duration were also observed, the difference in vaginal delivery rate did not reach statistical significance. This suggests that baseline delivery conditions and cervical maturity may partly influence the drug effect.^[18,19]^

Overall, this study suggests that misoprostol combined with oxytocin has potential value in promoting cervical ripening and improving the success rate of labor induction, particularly among primiparous women or those with unfavorable cervical conditions. These findings may provide useful clinical reference for optimizing induction protocols and improving maternal and neonatal outcomes.

It should be noted that this study was a single-center retrospective analysis, and the results may be influenced by selection bias and information bias. In addition, the limited sample size makes it difficult to fully reveal differences across subgroups. Moreover, this study did not conduct an in-depth evaluation of drug dosage, administration routes, or potential adverse effects. Therefore, future large-sample, multicenter, prospective randomized controlled trials are needed to further validate these findings and to comprehensively assess the safety and efficacy of the combined regimen, including its impact on long-term maternal and neonatal outcomes.

## Author contributions

**Conceptualization:** Zehua Zheng, Yang Feng, Wei Yin, Dongfei Guo, Qingran Kong, Xuefei Lu.

**Data curation:** Zehua Zheng, Yang Feng, Wei Yin, Dongfei Guo, Qingran Kong, Xuefei Lu.

**Formal analysis:** Zehua Zheng, Yang Feng, Wei Yin, Dongfei Guo, Qingran Kong, Xuefei Lu.

**Funding acquisition:** Yanli Pei, Qingran Kong, Xuefei Lu.

**Investigation:** Dongfei Guo, Yanli Pei, Xuefei Lu.

**Writing – original draft:** Yanli Pei, Xuefei Lu.

**Writing – review & editing:** Zehua Zheng, Wei Yin, Xuefei Lu.
